# Adolescent outcomes and opportunities in a Canadian province: looking at siblings and neighbors

**DOI:** 10.1186/1471-2458-14-506

**Published:** 2014-05-26

**Authors:** Leslie L Roos, Randy Walld, Julia Witt

**Affiliations:** 1 Manitoba Centre for Health Policy, Department of Community Health Sciences, Faculty of Medicine, University of Manitoba, 408-727 McDermot Avenue, Winnipeg, Manitoba R3E 3P5, Canada; 2 Department of Economics, Faculty of Arts, University of Manitoba, 554 Fletcher Argue Building, Winnipeg, Manitoba R3T 2 N2, Canada

**Keywords:** Child health, Sibling correlation, Life outcomes, Inequality persistence, Educational achievement, Teenage pregnancy, Health costs

## Abstract

**Background:**

Well-organized administrative data with large numbers of cases (building on linked files from several government departments) and a population registry facilitate new studies of population health and child development. Analyses of family relationships and a number of outcomes--educational achievement, health, teen pregnancy, and receipt of income assistance--are relatively easy to conduct using several birth cohorts. Looking both at means/proportions and at sibling correlations enriches our study of opportunity and well-being in late adolescence. With observational research possibly exaggerating the causal effects of risk factors, sibling comparisons involving individuals sharing both many family characteristics and many genes help deal with such criticisms.

**Methods:**

This paper uses a rich dataset from one Canadian province (Manitoba) covering a wide range of geographical areas (cities to rural regions). Influences on opportunity and well-being are analyzed looking at both means/proportions and sibling correlations. We measure a variety of outcomes that may reflect different causal influences. A creative application of linear programming advances the use of data on residential location.

**Results:**

Predicting educational achievement using available variables was much easier than predicting adolescent health status (R-square of .200 versus R-square of .043). Low levels of educational achievement, high levels of teenage pregnancy, and high sibling correlations outside Winnipeg and within Winnipeg’s lower income areas highlight inequalities across socioeconomic and geographic backgrounds. Stratifying our analyses by different variables, such as income quintiles, reveals differences in means and correlations within outcomes and across groups. Particular events--changes in mother’s marital status and in place of residence--were associated with less favorable outcomes in late adolescence.

**Conclusion:**

Our findings suggest a paradox: Canadian developmental outcomes through late adolescence appear quite similar to those in the United States, even though intergenerational mobility in Canada is closer to mobility in the Nordic countries than to that in the United States.

## Background

The growth of information-rich environments—well-organized administrative data with large numbers of cases building on multiple files, record linkage, and a population registry—has allowed extending studies of population health and human development. Analyses of family relationships and a number of important health and social outcomes are relatively easy to conduct using multiple birth cohorts.

Researchers have noted the need for greater insight into the relationship between health, education, and socio-economic status [1]. Sibling correlations provide an additional way (beyond comparing simple means and proportions) to describe disparities emerging in the early life course. Moreover, such correlations among health and social outcomes (health, education, teen pregnancy, and work force participation) “provide a broad measure of the overall importance of a wide range of factors common to the family, ranging from parental involvement to school and neighborhood quality” [2]. Sibling correlations vary among countries; this paper considers such variation within a province. Inequality of opportunity and low intergenerational mobility have also been linked to high sibling correlations [3,4]. A somewhat different literature has examined social and health factors during childhood. Parental socioeconomic status affects child health, which is related to future educational and labor market possibilities [5]; poor health status and consequently high health care costs may be more correlated among siblings and possibly neighbors. Childhood circumstances directly and indirectly influence adult health controlling for effort (decisions for which an individual is fully responsible) [6,7]. Human potential is not fulfilled when the possibilities vary so much among families and across environments.

One goal of this research is to provide “new descriptive facts” concerning disparities emerging early in the life course. This paper examines the following questions:

1) What are the relative sizes of sibling and neighbor correlations across a range of social and health variables?

2) What affects the magnitude of these correlations?

3) How does combining data on sibling correlations with more standard analyses broaden our perspectives on inequality?

This paper uses a rich dataset from one Canadian province (Manitoba) covering a wide range of geographical areas (cities to rural regions). Linear programming creatively uses residential location data to choose comparison groups by minimizing geographic distance between neighbors. Stratification by different variables, such as income quintiles, reveals differences in means and correlations within outcomes and across groups. Finally, we discuss a variety of measures that may reflect different causal influences and consider Canadian outcomes from a wider perspective.

### Comparing outcomes

What proportion of inequality in socioeconomic and health-related outcomes is attributable to specific family situations and to the communities that children grow up in? Siblings may be similar not only as a result of shared family background, but also due to such common factors as growing up in similar neighborhoods, going to the same school, sharing the same friends, and so on [8].

Separating family and neighborhood effects is important for understanding transmission mechanisms that affect intergenerational mobility. Outcomes among unrelated neighboring children are often contrasted with measured sibling correlations. Neighborhood effects appear to be relatively small: Solon et al. [9] found the correlation between neighboring children in educational attainment to be approximately 0.1 while more than 0.5 among siblings. Larger geographic distinctions (such as that between urban and non-urban areas) may be more important than neighborhood. For example, Page & Solon [10] showed most of the neighbor correlation to be explainable by simply growing up in an urban (as opposed to a nonurban) location rather than by which part of the city the child grew up in. In contrast, defining neighborhoods with relatively small boundaries tends to increase estimates of neighborhood effects, while length of exposure to a given neighborhood may also prove important [11-17].

Disentangling family and neighborhood effects on life course outcomes poses some challenges. Unmeasured family factors affecting both choice of neighborhood and child well- being may lead to apparent, but spurious, neighborhood effects [9,13,18,19]. Biases due to omitted variables, attrition, and measurement errors have complicated attempts to control for individual, family and neighborhood covariates [20-22]. The American Panel Study of Income Dynamics (PSID) is often used; its heterogeneous, nationally representative sample encompasses a wide range of state-level social services, school curricula, and economic circumstances [1,10]. More homogeneous data sets (for example Behrman & Taubman’s [23]) sample of white male veteran twins and their offspring) tend to underestimate the sibling correlation due to such samples’ lower variance than the general population [24].

Within-province analyses reduce some of the social differences among neighborhoods, while maintaining heterogeneity between families and neighborhoods. All Manitoba residents have access to the same health care system and social services. A provincial curriculum applies to all children (except for a few students under First Nations jurisdiction). Biases in loss to follow-up are minimal [25].

The measures chosen provide an opportunity to examine more than one domain of well-being simultaneously [26]. Analyses were conducted for a range of outcomes, including educational achievement (the Language Arts (LA) achievement index), health status (Aggregated Diagnosis Group (ADG) morbidity score), health care costs, teenage pregnancy, not being in grade 12 at the appropriate age, and receipt of income assistance. These variables were chosen due to a) their importance in the literature on education, health, and social policy, b) their availability in the data set, and c) their measurement characteristics [27,28]. Two of the measures (the Language Arts achievement index and the ADG morbidity score) were well validated indices.

## Methods

### Setting

Manitoba is reasonably representative of Canada as a whole, generally ranking in the mid-range of a series of indicators of health status, health care expenditures, and education [25,29]. In 2011, the provincial population was 1.208 million, and more than half (730,018) live in the Winnipeg Census Metropolitan Area, Canada’s eighth largest metropolitan area [30]. Located near the geographic center of Canada, Manitoba has a comparatively large aboriginal population (12.7%). The province provides relatively equal educational funding, with schools having more low-income families receiving more funding [31,32]. Manitobans score slightly below the Canadian average on standardized tests administered internationally (while Canadians do somewhat better than Americans) [30,33]. Canada’s safety net is more extensive than those in the United States and the United Kingdom [34,35]. Winnipeg has a substantial portion (over 8 per cent) of low income people, a figure below the median percentage for ten major Canadian cities studied in the 2000–2009 period [36]. The single-payer Canadian system tends to reduce disparities in health care access. Since “Canadian provinces and metropolitan areas had lower income inequality than US states and metropolitan areas”, Ross et al. [37] have suggested that the effects of income inequality on health ‘may be blunted’ by differences in the distribution of social and economic resources across the two countries.

### Linkage, sample, and follow-up

**Figure 1:**
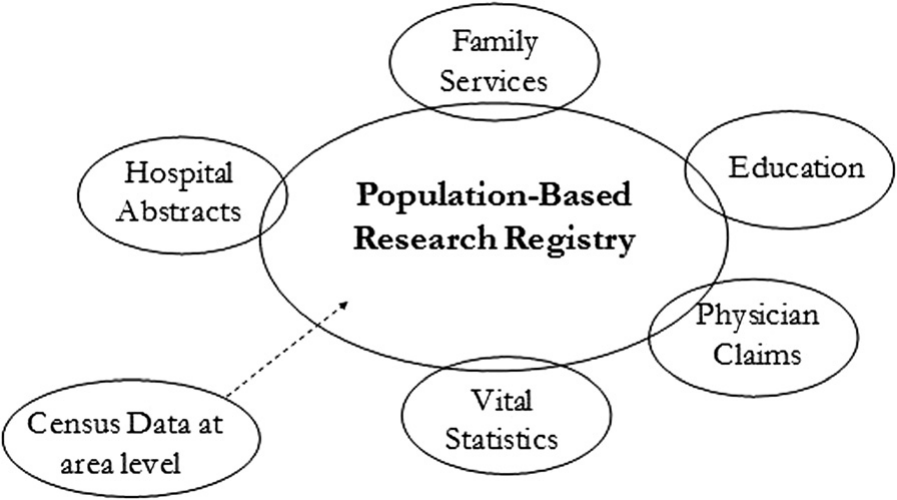
**Manitoba Centre for Health Policy research repository.** The research registry provides date of birth, sex, marital status, residential location (within Manitoba), date of entry in and departure from the province, and death information for essentially each Manitoba resident. To the extent possible, this information is compared with the Vital Statistics files. Information on each of the substantive files is provided in the Manitoba Centre for Health Policy website (http://umanitoba.ca/faculties/medicine/units/community_health_sciences/departmental_units/mchp/resources/repository/index.html).

Permission for use of the study data was obtained from the University of Manitoba Research Ethics Board, the Manitoba Health Information Privacy Committee, and the data providers (Manitoba Health, Manitoba Education, and Manitoba Entrepreneurship Training and Trade). A unique capacity to link different sources of data and provide a range of outcomes exists within Manitoba [38]. Figure 1 shows the organization of the data within this environment. The Population Health Research Data Repository at the Manitoba Centre for Health Policy (MCHP) is built from records processed by Manitoba Health to remove patient identifiers, such as name and address, while preserving the capacity to link records together to form individual histories. The repository is described more extensively elsewhere [39,40].

The sample includes approximately 75% of all children born in Manitoba in 1978 to 1982 and 1984 to 1987. The 1983 birth cohort is not included because Grade 12 provincial tests were not given in the school year 2000/2001 (when the 1983 birth cohort would be expected to be in Grade 12). Health, educational and income assistance outcomes are tracked up to fiscal year 2006.

The attrition rate of roughly 23% is from the original cohort covering over 98% of those born in the province. Migration out-of-province (primarily responsible for this attrition) was largely uncorrelated with several measures of health and socioeconomic status. As infants the group lost to followup is virtually identical to that remaining in Manitoba. Apgar scores (1 minute and 5 minute), gestational age, and birth weight (as well as mother’s age at first birth) are all very similar. Families leaving the province seem, on average, to be in the middle of the family formation process; the overall number of children is fewer and birth order is earlier than those remaining. Those lost to followup are less likely to be born to a married mother; a number of marriages occur later in the family formation process. After controlling for family fixed effects, “estimates of the impact of infant health on later outcomes among Manitoba residents do not appear to be biased from some fraction of our sample leaving the province” [25]. Childhood deaths provide a small amount of attrition. Children dying before age eight were much less healthy at birth; most of these deaths occurred within the first year of life [41].

Record linkage of files from the Ministry of Education (the education data) and the Ministry of Entrepreneurship, Training and Trade (the income assistance data) with the registry allowed identification of cohort members in the province but not enrolled in school [38]. Linkage quality was high; for example, only 2.8% of all students enrolled in 2002 could not be linked to the December 2001 registry [42].

### Data quality

Each substantive file is checked against the registry for accuracy of the identifiers and for such particular information as date of in-hospital death [39]. The research registry, coordinated with Vital Statistics files, provided information on place of residence using a six-digit postal code, as well as dates of arrival and departure (births, deaths and moves) for any date since 1970 [43]. Time-sensitive data elements (place of residence, family composition) are updated using “snapshot” registries provided every six months.

This paper uses siblings and non-related children from neighboring families from the nine Manitoba cohorts born in 1978 – 1982 and 1984 – 1987. Twins were not included because of the difficulty of matching twin pairs in the same neighborhood; across a number of measures, the correlations between twins are higher than those between siblings [25,44]. Only non-overlapping pairs of same-sex siblings and neighbors were used, and only two sisters or brothers from a given family were selected for each same-sex analysis. Using just one sibling pair from each family gives equal weight to families, regardless of the total number of children in the family. Correlation estimates may change with different weighting schemes, though more equal weighting schemes tended to produce better results [9]. Analyses using all available children have produced results very similar to those just pairing siblings [45]. The sampling also ensured that Manitoba residence was maintained over the entire period and that the sampled children were at least half siblings. Correlations based on all sibling pairs (including half siblings) differed little from those when only full siblings were included. The mean age difference between same-sex siblings was 3.1 years, and between unrelated neighbors 2.7 years.

### Defining neighborhoods

**Figure 2:**
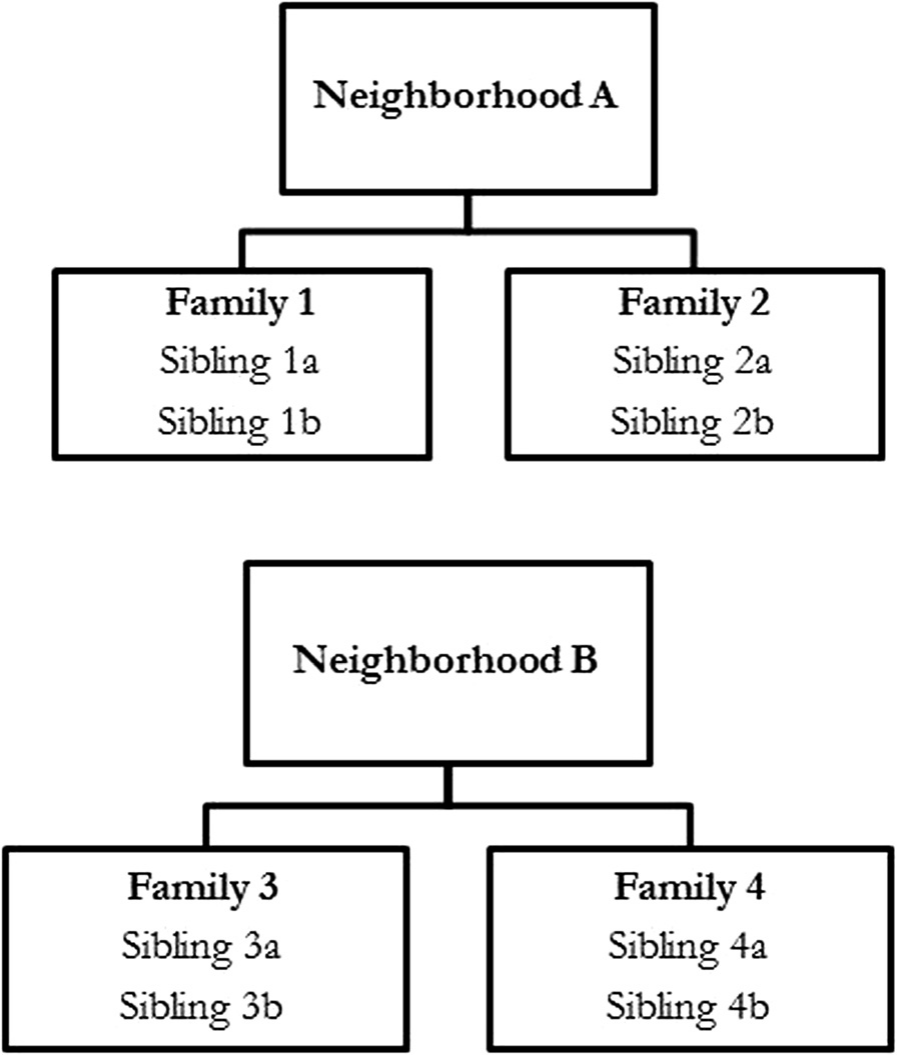
**Sibling - neighborhood designs.** Within each family, siblings a and b are compared. Within each neighborhood, the two appropriate families are compared: the two sibling a’s with each other and the two sibling b’s with each other.

Canadian postal codes tend to represent quite small areas in cities, with several postal codes typically contained within a Statistics Canada census dissemination area. Using postal codes to help define neighborhoods is important, given recent arguments emphasizing local comparisons [46]. Dissemination areas (from the 2001 census) are usually assigned to be between 400 and 700 persons and provide descriptive data. The residential postal code where the older sibling lived at age 17 designated each sibling pair. Of same-sex sibling pairs, 90.3% could be compared with a similar pair of unrelated neighbors in the same census dissemination area (Figure 2). Including those inside and outside of Winnipeg, 63.5% of the groups (*N* = 9,424) resided within the same postal code area. With odd numbers of families in a postal code area, the ‘nonmatched’ family was a potential match for another such family having a different postal code within the same census area. A linear program used the simplex method, pairing families to minimize the total distance between centroids of potential matches within the census area (36.5% of the sample, *N* = 5,412). Thus, neighborhood is defined as either a particular postal code area (with a pair of families having the same postal code) or two postal codes close to each other in the same dissemination area (with each paired family having a different residential postal code). Given an odd number of families in a census area, the linear program eliminated that family whose place of residence proved most difficult to pair with another. Calculations used PROC LP in SAS/OR (version 9.2).

With rural dissemination areas geographically larger and small towns often having a single postal code, neighbors outside Winnipeg are likely to live farther away from each other than their urban counterparts. Our method based on postal codes and linear programming produced closer ‘neighbors’ than did the traditional method of randomly selecting ‘neighbors’ from residents of the same census area. Within Winnipeg, the percentage of the sample in the same postal area was smaller using the traditional method (52.1% versus 63.5%). Of those ‘neighbors’ not in the same Winnipeg postal area, the mean difference between their area centroids was 0.33 km by the traditional method, compared with 0.21 km for their counterparts assigned by linear programming. Improvements for the areas outside Winnipeg were much smaller. Neighbor correlations generated by the traditional random selection method are available from the corresponding author.

Both Canadian and American data show considerable persistence in neighborhood environments [15]. 56% of the nine-year birth cohort remaining in the province had no residential moves between the ages of 8 and 17.5, while another 24% moved only once (Table 1). 73.7% of those in these birth cohorts did not change postal codes over the 1991–1995 period. The log mean income of cohort members’ 1991 Manitoba census area was highly correlated with the log mean income of their 1992 census area (correlation = 0.937) and with the 1995 census area (correlation = 0.840). With cohorts and their parents five years older, 79.7% of the cohorts did not move over the 1996 – 2000 interval.

**Table 1:** Characteristics of Manitoba birth cohorts (1978–1982, 1984–1987) and study sample

**Variable**	**Format**	**Birth cohort members**	**Siblings in families with at least one same-sex pair**	Study sample of same-sex sibling pairs*
Place of Residence and Age				
Winnipeg	Percent	47.7	40.9	39.5
Age at Jan 1, 2000	Mean (SD)	16.8 (3.02)	16.9 (2.85)	16.8 (2.83)
Family Variables				
Maternal age at first birth	Mean (SD)	23.2 (4.55)	22.7 (4.24)	22.9 (4.25)
Number of residential moves between child’s age 8 and 17.5	Mean (SD)	0.87 (1.45)	0.84 (1.49)	0.79 (1.40)
Birth order	Mean (SD)	2.02 (1.16)	2.35 (1.36)	2.35 (1.35)
Number of children in family	Mean (SD)	3.05 (1.55)	3.76 (1.80)	3.63 (1.74)
Mother married at birth of child	Percent	81.5	84.7	87.1
Months in which family received income assistance between child’s age 8 and 17.5	Mean (SD)	5.07 (19.11)	4.98 (18.96)	4.43 (17.89)
Continuous Outcomes				
Language Arts Index (scaled logit)	Mean (SD)	0.00 (1.00)	-0.08 (1.03)	-0.04 (1.01)
Health Status (ADG Morbidity Score) (age 12–17)	Mean (SD)	9.69 (6.45)	9.08 (6.26)	9.11 (6.24)
Hospital and Physician Costs (age 12–17, C$1993)	Mean (SD)	1,158 (4,661)	1,164 (5,315)	1,137 (5,091)
Binary Outcomes				
Not in Grade 12 at Appropriate Age	Percent	28.7	32.4	30.6
Teenage Pregnancy (females to age 19)	Percent	13.1	14.4	13.5
Received Income Assistance (age 18–19)	Percent	5.7	5.8	5.3
Number of Boys		54,056	20,391	15,660
Number of Girls		51,382	18,649	14,012
Number of Families		71,126	16,060	14,502

* 334 families contributed a sibling pair of both sexes, so the total number of pairs used in the sibling/neighborhood analysis was 14,836.

The calculations for "maternal age at first birth", "number of children in family", and "birth order" were based on files which go back to 1970 for the mother. This leads to a slight overestimate of maternal age at first birth and a slight underestimate of the other two variables (since a few children will have been born before 1970). Twins were excluded from birth cohorts and samples. Birth cohort members included those born in and remaining in the province between birth and age 17.5.

Further analyses divide the Statistics Canada census dissemination areas inside Winnipeg and outside Winnipeg into five equal-sized groups ranked from lowest to highest income (based on mean household income within each dissemination area). Statistics Canada did not take into account the number of individuals in each household. The ordering of these areas is quite stable, with correlations about 0.85 over five-year census intervals. Dividing the sibling samples according to residential income quintiles generated from the whole population has both advantages and disadvantages. With families tending to move up economically during the child-rearing years, the lowest income areas (Q1) have disproportionately higher numbers of elderly and recent immigrants. In the Winnipeg same-sex sibling sample, Q1 neighborhoods have just 1,436 young people while the highest income areas (Q5) have 3,304. On the other hand, with relatively few adolescents in the most affluent neighborhoods behind in school (18.6%) or having children as teenagers (6.3% of the girls), the larger Q5 sample ensures having enough individuals in this category.

### Outcome measures

#### ***Educational achievement***

Two measures of educational achievement were used. The first measure, the Language Arts achievement index, was generated from provincial standards tests taken in Grade 12 and treated as a continuous outcome measure. These tests contribute 30% to the final course grade; scoring 50% or higher is a passing mark. Scores are recorded in 5 percentage point categories (19 in total) in the year that most students write the test. For those not writing, the multi-file data provide considerable additional information. Additional categories of educational achievement were ranked below the lowest score attained by those writing the test. These additional categories are (from highest to lowest rank, corresponding with the probability of high school graduation): absent (around 1% of each birth cohort sample); in Grade 12 but not tested (8%); in Grade 11 or lower (19%); not enrolled (2%); withdrawn from school (10%). Following Mosteller & Tukey [47] and Willms [48], a standardized score for each individual was computed by assuming an underlying logit distribution, divided into pieces according to the percentage of cohort members in each category. Scores were calculated separately for each birth cohort because of small changes in the categories available and in the percentage distribution each year. In a typical year, the highest scorers reached an index score of 2.96, while those withdrawn from school were given a score of -1.84. The logit transformation produces an index with an overall mean of zero and a standard deviation of one. The point biserial correlation between this Language Arts index and the students’ probability of graduating in four years (available from a subsample of two birth cohorts) is 0.54 (*p* < 0.0001) [38]. For sensitivity testing, runs were repeated using only observations having scores from the provincial standards tests (eliminating the bottom 20 percent of the distribution) (this work is summarized in Appendix 1). Linking the Manitoba data with Statistics Canada’s Community Health Survey has shown scores on the Language Arts achievement index to predict the probability of postsecondary education and its completion [49].

The second measure of educational achievement was based on enrolment records: not attaining Grade 12 by age 17 could indicate that a student entered school late, dropped out, or was held back in a grade at least once. Approximately 3% of children start Kindergarten a year late; they tend to be born in November and December. Matching the birth cohorts to enrolment records for the full nine year sample until age 17 generated an indicator of the risk of having a low level of education attainment. 28.7% of all birth cohort members were not in Grade 12 at the appropriate age.

### Health status

With over 90 per cent of the Manitoba population contacting a physician over a two-year period (averaging more than four visits annually), the use of administrative data to estimate health status is well-established [25]. Aggregated Diagnosis Group (ADG) scores (part of the Adjusted Clinical Group (ACG) case-mix system) provided a measure of the burden of morbidity, with higher scores indicating a higher burden (i.e. more co-morbidities) [50]. This diagnosis-based, case-mix methodology uses hospital discharge abstracts and physician claims to describe a population’s health care utilization. This measure was developed at Johns Hopkins and validated with administrative data from Manitoba and at least two other Canadian provinces [51-53]. The number of ADGs was calculated from administrative data for each year the child was between the ages 12 and 17; this excludes routine visits (for immunizations, for example) before the age of 12 [25]. This index is one of several claims-based measures similarly correlated with mortality [54]. The mean morbidity score of all cohort members was 9.69.

### Health care costs

Health care costs provide another way to estimate health status. Earlier work has used the number of physician visits and the number of hospital stays to estimate health status [25,55,56]. A cost measure picks up both multiple physician visits (as often accompany chronic disease) and hospital stays (typically indicating a more serious condition). Hospital and physician costs were aggregated for each individual from age 12 to 17. Physician costs were largely through fee-for-service; direct information on physician visits was available in the database (excluding a few patient visits outside the province and some visits to salaried physicians) [57]. The costs of inpatient hospital care and day surgery procedures were estimated by applying the Manitoba average cost per weighted case to each discharge [58]. Costs were allocated to individual patients and then aggregated. Some costs were not captured, either because they were not attributable to specific patients (the costs of running some hospital clinics) or because patient-specific data are lacking. These include costs attributable to northern nursing stations, blood products, or CancerCare Manitoba [55]. The mean hospital and physician cost of those age 12 to 17 was $1,158. Health status and health care cost measures reflect somewhat different aspects of health and illness. A child with a single serious illness might experience a long hospital stay and several expensive procedures. In this case, health costs would be high with the health status measure (relatively) low.

### Teenage pregnancy

Jutte et al. [59] have emphasized the risks of adolescent motherhood for children’s social, educational, and medical outcomes. Teenage pregnancy tends to be repetitive (daughters of teenage mothers are more likely to become teenage mothers themselves) and a significant indicator of poorer socioeconomic outcomes later in life [60]. The measure includes all teenage pregnancies that ended in births, stillbirths or abortions (spontaneous or therapeutic). The same observation window is used for each teenager in the sample. The teenage pregnancy rate in the 1978–1982 and 1984–1987 birth cohorts was 13.1%, while the birth rate (i.e. only pregnancies resulting in birth) was 8% before the age of 19 in the 1982 and 1984–1989 birth cohort.

### Income assistance

The probability of receiving income assistance and the correlations between siblings may result from several factors—such as a family’s (lack of) emphasis on education and local employment opportunities. Information on whether members of the birth cohorts received income assistance was available up to March 2007, providing a 15-month follow-up for the entire sample (starting at age 18). Thus, the maximum exposure to income assistance eligibility possible with our data is used to ensure that all individuals were observed for the same length of time. Sensitivity testing with a longer follow-up period but a more restricted sample (using just the 1978 – 1982 birth cohorts) produced very similar results. 5.7% of the birth cohort members received income assistance in the 15 months after age 18.

### Statistical model

The estimation employs a mixed model in which the data are permitted to show correlation and nonconstant variability [61]. In order to disentangle the different sources of variation, clusters are specified at the family level (two same-sex siblings form a sibling pair) and at the neighborhood level (two sibling pairs from different families residing in the same dissemination area). The data are fit using restricted maximum likelihood (REML) since they are assumed to have a Gaussian distribution. REML only maximizes the likelihood of the data for the random effects, hence “restricted” ML. Continuous outcomes (Language Arts index, health status, hospital & physician costs) were estimated using a linear mixed model; the PROC MIXED model in SAS computes the standard errors of the variance components. A 95% confidence interval was constructed as +/-1.96 standard error of the variance component. Binary outcomes (Grade 12 at appropriate age, teenage pregnancy, income assistance) were estimated using a non-linear (logit) mixed model, (the NLMIXED procedure in SAS). The intraclass correlation (ICC) parameter and a 95% confidence interval are estimated by the model. Appendix 2 provides further details on the modeling.

Additionally, neighbor correlations were adjusted using fixed effects since families in similar neighborhoods may share characteristics possibly explaining some of the correlation [9]. In their model, sibling covariance is the sum of shared family variance, shared neighborhood variance, and twice the covariance between family and neighborhood factors [9]. Neighbor covariance is the sum of the covariance in family backgrounds among neighboring children, shared neighborhood variance, and twice the covariance between family and neighborhood factors. Sibling and neighbor covariance differ only in the first terms (shared family variance in the sibling covariance, and covariance in family backgrounds among neighboring children in the neighbor covariance), while the other two terms are the same. Neighbor correlations are upward biased for two reasons. First, sharing similar backgrounds with neighbors (the covariance between family and neighborhood factors) is not a true neighborhood effect because advantaged families sort themselves into advantaged neighborhoods [9]. Second, the entire covariance between family and neighborhood factors (the first term in the neighbor covariance) is attributed to neighborhood effects in calculating the correlation. Given ambiguity in allocating covariance, the neighbor correlation appears overly generous in estimating the possible influence of the shared environments, including both measured and unmeasured variables [9,62]. The bound on the neighbor correlations can be tightened by subtracting measured family variables from the shared family background component using the residuals from a regression of outcome measures on known family characteristics and correlating these across neighboring children [62]. Implementing this approach adjusted neighbor correlations for the continuous outcomes. However, since residuals cannot be generated for binary outcomes, fixed (family) effects were directly included in the non-linear model [9] used to calculate the correlations.

By absorbing some of the heterogeneity, the family effects reduce the neighbor correlations. Available family predictors included in the adjusted model are maternal age at first birth; number of residential moves between age 8 and 17.5; birth order; number of children in the family; mother’s marital status at birth; and family receipt of income assistance between age 8 and 17.5. These characteristics were based on information associated with the older sibling. Generally, values changed little when younger sibling information was used. (Birth order values increased by one!). Regression analysis measured the impact of each of these predictors on the outcome measure and the overall explanatory power of each of these models.

## Results and discussion

### Representativeness

Table 1 compares three groups remaining in Manitoba through 17.5 years of age: all those in the birth cohort, siblings in families with at least one same-sex pair, and siblings sampled in this study. Given larger families in rural areas, the study undersamples Winnipeg children. Compared to all birth cohort members, the sampled family is slightly less likely to have received income assistance and to have changed residences; the mother is more likely to have been married at the time of birth of each child. The sampled children have slightly lower health care costs between the ages of 12 and 17. These differences are largely due to sample recruitment from those families with at least one same-sex pair of children.

### Siblings and neighbors

Table 2 lists the six variables noted above, their category frequencies and their coefficients in the mixed-effects regression predicting the Language Arts achievement index and health status (ADG morbidity score) for all 105,438 adolescents. Whether or not the child’s family received income assistance (rather than the number of months of assistance) was used because of the skewed distribution of this variable. Compared with research using the well-known Panel Study of Income Dynamics (PSID), the six family variables predicted scores on the Language Arts Index (*R*^*2*^ = 0.200) relatively well [63]. Predicting health status was much more difficult, generating an *R*^*2*^ of just 0.043. Maternal age at first birth (particularly the younger ages) was less likely to be statistically significant vis-à-vis health status. These models are used to adjust the neighbor correlations. Table 3 summarizes the effects of the six variables across outcomes; analyses using all birth cohort members and the study sample of same-sex sibling pairs show similar results. Continuous and binary outcomes are not directly comparable; *R*^*2*^ is reported for the continuous outcomes and the C statistic for the binary outcomes [64]. Overall predictability depends on the measure chosen. The binary outcomes (not in Grade 12 at appropriate age, teenage pregnancy, and receipt of income assistance) have a “social” component to them and show moderate predictability. A C statistic of 0.5 implies that the predictive power of the model is equivalent to “chance”; 1.0 is perfect prediction.

**Table 2:** Predicting educational achievement and health status

		**Language arts index**		**Health status (ADG morbidity score)**	
		**R-square: .200**		**R-square: .043**	
**Predictors**	**%**	**Beta**	**p**	**Beta**	**p**
Maternal age at first birth: 15 and under	1.83%	-0.237	<.0001	0.154	0.3173
16–17	7.71%	-0.187	<.0001	-0.004	0.9671
18–19	13.46%	-0.113	<.0001	-0.066	0.3711
20–21 (reference)	15.13%	0.000	--	0.000	--
22–23	16.11%	0.138	<.0001	0.090	0.1991
24–25	15.53%	0.259	<.0001	0.238	0.0008
26 and over	29.80%	0.356	<.0001	0.332	<.0001
Missing	0.43%	-0.339	<.0001	-0.492	0.2956
Group p-value (7 df)			<.0001		<.0001
Number of residential moves between age 8 and 17.5:					
Zero (reference)	56.22%	0.000	--	0.000	--
One	24.00%	-0.026	0.0001	0.386	<.0001
Two	9.79%	-0.117	<.0001	0.730	<.0001
Three	4.54%	-0.190	<.0001	1.147	<.0001
Four	2.33%	-0.245	<.0001	1.422	<.0001
Five or more	3.12%	-0.297	<.0001	2.432	<.0001
Group p-value (5 df)			<.0001		<.0001
Birth order	2.02^a^	-0.044	<.0001	-0.038	0.0777
Number of children in family	3.05^a^	-0.104	<.0001	-0.670	<.0001
Married at birth of child	81.51%	0.306	<.0001	0.199	0.0005
Missing marital status	0.70%	0.166	0.0015	-0.283	0.4435
Not on Income Assistance age 8 to 17.5	88.87%	0.341	<.0001	-1.910	<.0001
N		105,438		105,438	

^a^Mean value.

The full sample of children born from 1978 to 1987 (except those born in 1983) was included in this table.

**Table 3:** Predicting outcomes using six family variables

	All birth cohort members*	Study sample of same-sex sibling pairs**
**Continuous outcomes (R-square statistic)**		
Language arts index	0.200	0.234
Health status (ADG morbidity score)	0.043	0.055
Hospital and physician costs	0.017	0.021
**Binary outcomes (C Statistic**)^**†**^		
Not in grade 12 at appropriate age	0.749	0.774
Teenage pregnancy*	0.752	0.765
Received income assistance (age 18–19)	0.824	0.823

*The 1978–1982, 1984–1987 birth cohorts included 105,438 individuals. The n for “teenage pregnancy” was 51,382 females.

**The birth cohorts in the study sample of same-sex sibling pairs included 29,672 individuals. The n for “teenage pregnancy” was 14,012 females.

^†^The C statistic represents the area under the receiver operating characteristic curve.

Mixed effects regressions were used for both the continuous and binary outcomes [65].

### Inside and outside Winnipeg

Large, statistically significant differences in mean scores, particularly in educational achievement, were found between those inside and outside Winnipeg (Table 4). Table 5 presents the high sibling and low neighbor correlations; adjustment using the measured family variables further reduces neighbor correlations. Much of the apparent neighborhood impact on socioeconomic outcomes appears due to the similarity among neighbors. Inside Winnipeg and outside Winnipeg sibling correlations differ significantly for the Language Arts Index with re-expressed values (p < 0.01), timely school completion (p < 0.01), Hospital and Physician Costs (P < 0.01) and teenage pregnancy (p < 0.05). Neighbor correlations, both adjusted and unadjusted, do not differ significantly from each other, except for teenage pregnancy in the unadjusted model (p < 0.01). Generally, neighbor correlations tend to be higher among those outside Winnipeg, while sibling correlations display no such pattern.

**Table 4:** Outcomes inside and outside Winnipeg

	**Winnipeg**	**Outside Winnipeg**
**Variables**	**N = 11,728**	**N = 17,944**
Continuous outcomes (mean)		
Language arts index	0.176 (0.159–0.194)	-0.189 (-0.204–-0.174)
Health status (ADG morbidity score)	10.24 (10.12–10.35)	8.38 (8.29- 8.47)
Hospital and physician costs	$1,160 ($1,061–$1,260)	$1,121 ($1,051–$1,191)
Binary outcomes (proportion)		
Not in grade 12 at appropriate age	.206 (.199–.213)	.372 (.365–.379)
Teenage pregnancy	.129 (.120–.138)	.139 (.131–.146)
Received income assistance (age 18–19)	.063 (.059–.067)	.047 (.044–.050)

The N refers to the number of individuals (twice the number of same-sex pairs matched with their neighbors) in the study sample. The N for “Teenage Pregnancy” was 5,508 and 8,504 females for Winnipeg and Outside Winnipeg respectively.

Confidence intervals (95%) are in parentheses.

**Table 5:** Sibling and neighbor correlations inside and outside Winnipeg

	**Winnipeg**	**Outside Winnipeg**
**Variables**	**N = 11,728**	**N = 17,944**
Sibling correlations		
Continuous outcomes		
Language arts index^†^	0.449 (0.429–0.469)	0.575 (0.561–0.589)
Health status (ADG morbidity score)	0.475 (0.455–0.495)	0.467 (0.451–0.483)
Hospital and physician costs^†^	0.321 (0.298–0.344)	0.260 (0.241–0.279)
Binary outcomes		
Not in grade 12 at appropriate age^†^	0.594 (0.560–0.628)	0.650 (0.627–0.673)
Teenage pregnancy*	0.467 (0.394–0.540)	0.576 (0.528–0.625)
Received income assistance (age 18–19)	0.637 (0.586–0.689)	0.651 (0.606–0.697)
Neighbor correlations (unadjusted)		
Continuous outcomes		
Language arts index	0.043 (0.017–0.069)	0.038 (0.017–0.059)
Health status	0.011 (-0.015–0.037)	0.019 (-0.002–0.040)
Hospital and physician costs	0.019 (-0.007–0.045)	0.037 (0.016–0.058)
Binary outcomes		
Not in grade 12 at appropriate age	0.316 (0.271–0.360)	0.316 (0.286–0.346)
Teenage pregnancy^†^	0.186 (0.101–0.271)	0.368 (0.308–0.428)
Received income assistance (age 18–19)	0.331 (0.256–0.407)	0.350 (0.280–0.420)
Neighbor correlations (adjusted)		
Continuous outcomes		
Language arts index	0.016 (-0.010–0.042)	0.030 (0.009–0.050)
Health status	0.008 (-0.018–0.034)	0.011 (-0.010–0.032)
Hospital and physician costs	0.020 (0.000–0.040)	0.031 (0.011–0.052)
Binary outcomes		
Not in grade 12 at appropriate age	0.101 (0.045–0.158)	0.105 (0.069–0.140)
Teenage pregnancy	0.037 (-0.063–0.138)	0.120 (0.045–0.194)
Received income assistance (age 18–19)	0.083 (-0.022–0.189)	0.182 (0.089–0.226)

The N refers to the number of individuals (twice the number of same-sex pairs matched with their neighbors) in the study sample. The N for “Teenage Pregnancy” is given in Table 4.

Confidence intervals (95%) are in parentheses.

Statistically significant differences: ^†^p < 0.01, *p < 0.05.

### Income quintiles

Residence in Winnipeg’s lower income areas was associated with poorer performance on the Language Arts achievement index, higher probabilities of not being in Grade 12 at the appropriate age, and higher probabilities of teenage pregnancy (Table 6). Four of the six sibling correlations in Winnipeg showed siblings in lower income residences to be much more likely to have similar outcomes.

**Table 6:** Means, proportions, and sibling correlations by income quintile of residence (Winnipeg)

	**Quintile of residence**				
**Outcomes**	**Q1**	**Q2**	**Q3**	**Q4**	**Q5**
Continuous outcomes (mean)					
Language arts index^†^	-0.49	-0.04	0.16	0.36	0.57
Health status (ADG morbidity score)	10.06	10.15	10.23	10.28	10.32
Hospital and physician costs	$1,231	$1,216	$1,184	$1,104	$1,140
Binary outcomes (Proportion)					
Not in grade 12 at appropriate age^†^	.524	.298	.210	.132	.087
Teenage pregnancy^†^	.309	.197	.122	.081	.063
Received income assistance (age 18–19)^†^	.204	.104	.063	.024	.017
Sibling correlations					
Continuous outcomes					
Language arts index^†^	0.540 (0.457–0.623)	0.468 (0.395–0.541)	0.377 (0.311–0.443)	0.338 (0.286–0.390)	0.302 (0.252–0.352)
Health status (ADG morbidity score)	0.493 (0.411–0.575)	0.466 (0.393–0.539)	0.475 (0.407–0.543)	0.461 (0.408–0.514)	0.484 (0.430–0.538)
Hospital and physician costs†	0.372 (0.294–0.450)	0.357 (0.287–0.427)	0.294 (0.230–0.358)	0.305 (0.254–0.356)	0.285 (0.235–0.335)
Binary outcomes					
Not in grade 12 at appropriate age^†^	0.672 (0.594–0.751)	0.544 (0.454–0.634)	0.471 (0.380–0.574)	0.391 (0.294–0.488)	0.285 (0.160–0.411)
Teenage pregnancy*	0.516 (0.365–0.667)	0.377 (0.217–0.537)	0.283 (0.086–0.479)	0.367 (0.187–0.548)	0.220 (0.000–0.458)
Received income assistance (age 18–19)	0.497 (0.383–0.612)	0.531 (0.404–0.658)	0.636 (0.512–0.760)	0.533 (0.357–0.708)	0.572 (0.383–0.761)
**N (except as noted)**	**1,436**	**1,756**	**2,032**	**3,196**	**3,304**

The N for “Teenage Pregnancy” ranged from 644 to 1,548 females.

Trend in continuous outcomes estimated by linear regression; in binary outcomes by Cochrane-Armitage test.

Trend in correlations estimated in PROC NLIMIXED.

*p for trend <0.05, ^†^p for trend <0.01.

The educational measures highlight regular, dramatic changes in both outcomes and sibling correlations. Plotting outcomes for sibling 1 and sibling 2 against each other illustrates a relationship at low levels of income, but much less so at high income levels. The higher correlation at low levels of income around a lower mean value suggests the greater persistence of poorer outcomes between siblings in lower income quintiles. The correlations for the Language Arts index decreased regularly from 0.540 in the lowest income neighborhoods to 0.302 in the highest; those for ‘not being in grade 12 at the appropriate age’ decreased from 0.672 to 0.285. Teen pregnancy varies substantially with residential income quintile; sibling correlations demonstrate a somewhat irregular trend. Reliance on social assistance drops dramatically with income quintile of residence while sibling correlations appear stable. Young people are generally healthy; both the health measures and relevant sibling correlations are fairly stable with relatively few trends (although correlations in hospital and physician costs are statistically significant). Finally, means and correlations generated outside Winnipeg showed no regular trends, perhaps reflecting the weaker relationship between individual and area household income outside Winnipeg and the variety of areas in rural Manitoba.

### Residential mobility and changes in marital status

Although the more dramatic differences are associated with income quintiles, other family circumstances and events affect both outcomes and sibling correlations. Analysis by sibling age difference generated several statistically significant findings but trends were difficult to ascertain (tables available from the corresponding author). Residential mobility and changes in mother’s marital status were both associated with less favorable outcomes. Residential mobility was based on the experience of the older sibling to assure counting family moves which affect both siblings. This avoids assigning a (family) move to an older sibling who after age 18 may have a very different pattern of residential mobility. Residential mobility had little effect on the sibling correlations (Table 7), with significant differences between zero moves and one move only for the LA index (*p* < 0.01) and timely school completion (*p* < 0.05). Differences between zero moves and two or more moves were found only for income assistance receipt (*p* < 0.01).

**Table 7:** Means, proportions, and sibling outcomes by residential mobility

	**Residential mobility**					
	**Zero moves**		**One move**		**Two or more moves**	
**Outcomes**	**N = 16,914**		**N = 7,184**		**N = 5,574**	
Continuous outcomes (mean)						
Language arts index	0.078	(0.063–0.094)	0.089	(0.066–0.112)	-0.352	(-0.377–-0.327)
Health status (ADG morbidity score)	8.686	(8.595–8.776)	9.389	(9.245–9.532)	10.064	(9.833–10.244)
Hospital and physician costs	$1,052	($984–$1,121)	$1,094	($961–$1,228)	$1,446	($1,299–$1,594)
Binary outcomes (proportion)						
Not in grade 12 at appropriate age	0.274	(0.267–0.281)	0.265	(0.255–0.275)	0.458	(0.445–0.471)
Teenage pregnancy	0.101	(0.094–0.107)	0.110	(0.100–0.120)	0.267	(0.250–0.284)
Received income assistance (age 18–19)	0.030	(0.027–0.032)	0.042	(0.037–0.047)	0.139	(0.130–0.148)
Sibling correlations						
Continuous outcomes						
Language arts index	0.560	(0.545–0.575)	0.500	(0.475–0.525)	0.531	(0.504–0.558)
Health status (ADG morbidity score)	0.483	(0.467–0.500)	0.480	(0.455–0.505)	0.470	(0.441–0.500)
Hospital and physician costs	0.287	(0.268–0.307)	0.276	(0.246–0.307)	0.283	(0.249–0.317)
Binary outcomes						
Not in grade 12 at appropriate age	0.655	(0.632–0.679)	0.604	(0.563–0.644)	0.614	(0.572–0.656)
Teenage pregnancy	0.524	(0.462–0.584)	0.434	(0.337–0.532)	0.471	(0.392–0.550)
Received income assistance (age 18–19)	0.661	(0.606–0.717)	0.587	(0.503–0.671)	0.549	(0.487–0.612)

For Outcomes:

Statistically significant differences between Zero Moves and One Move: Health Status, Received Income Assistance (p < 0.01).

Statistically significant differences between Zero Moves and Two or More Moves: All outcomes (p < 0.01).

For Correlations:

Statistically significant differences between Zero Moves and One Move: LA Index and Not in Grade 12 at Appropriate Age (p < 0.01).

Statistically significant differences between Zero Moves and Two or More Moves: Received Income Assistance (p < 0.05).

Almost one-third of the sample experienced a change in mother’s marital status (due to separation, to divorce or death, or to a single parent entering a new relationship) by age 17 (Table 8). Siblings will experience this change at different ages and may adapt differently. Sibling correlations are lower among these children for all outcomes except health status, differing significantly for the LA index (*p* < 0.01), timely school completion (*p* < 0.01) and teenage pregnancy (*p* < 0.05).

**Table 8:** Means, proportions, and sibling correlations by change in mother’s marital status

	**Change in marital status of mother by age 17 of oldest child**			
	**No changes**		**At least one change**	
**Outcomes**	**N = 20,082**		**N = 9,590**	
Continuous outcomes (mean)				
Language arts index^†^	0.095	(0.081–0.110)	-0.183	(-.202–-0.164)
Health status (ADG morbidity score)^†^	8.833	(8.748–8.918)	9.656	(9.529–9.783)
Hospital and physician costs^†^	$1,002	($953–$1,050)	$1,396	($1,255–$1,537)
Binary outcomes (proportion)				
Not in grade 12 at appropriate age^†^	0.262	(0.256–0.269)	0.391	(0.381–0.400)
Teenage pregnancy^†^	0.101	(0.095–0.107)	0.199	(0.188–0.210)
Received income assistance (age 18–19)^†^	0.035	(0.032–0.038)	0.088	(0.083–0.094)
Sibling correlations				
Continuous outcomes				
Language arts index^†^	0.561	(0.547–0.575)	0.514	(0.494–0.534)
Health status (ADG morbidity score)	0.476	(0.461–0.492)	0.490	(0.469–0.511)
Hospital and physician costs	0.287	(0.269–0.306)	0.280	(0.255–0.306)
Binary outcomes				
Not in grade 12 at appropriate age^†^	0.679	(0.658–0.700)	0.563	(0.529–0.597)
Teenage pregnancy*	0.555	(0.500–0.609)	0.451	(0.386–0.516)
Received income assistance (age 18–19)	0.655	(0.606–0.704)	0.603	(0.552–0.654)

For Correlations: Statistically significant differences: ^†^p < 0.01, *p < 0.05.

## Conclusions

We have merged administrative data from different government departments, moving beyond health into educational and social analyses. Means/proportions and sibling correlations provide somewhat different views, highlighting the potential of information-rich environments.

Despite Canada’s greater mobility in intergenerational earnings than in many OECD countries [66], sibling correlations are high and neighbor correlations low across our education, health, and labor force participation measures. The part of the province and city in which a child grows up seems particularly important. Differing possibilities across socioeconomic and geographic groups are highlighted by low levels of educational achievement, high levels of teenage pregnancy, and high sibling correlations outside Winnipeg and within Winnipeg’s lower income areas. Such data on means/proportions and correlations suggest a ‘double whammy’ affecting socioeconomic mobility: the overall possibilities for improved well-being are relatively low and within-family dynamics provide further hindrance.

Circumstances matter. These sibling correlations indicate more widespread availability of opportunities for the affluent. Gradients for the education-related measures parallel those noted for intelligence and educational achievement in American twin studies [67,68]. Such gradients do not appear for the health-related variables; in late adolescence, individual health (at least according to our measures) seems relatively independent of family income.

Many Canadian outcomes through late adolescence appear roughly similar to those in the United States. Sibling correlations in educational attainment are relatively high—in the 0.5 [9] to 0.6 range [2]. American correlations were considerably lower for health-related measures [2]. Several lines of evidence have suggested contextual factors (income inequality, neighborhood social environment) to be more important determinants of health in the United States and the United Kingdom than in Canada and other developed countries [16,17,20,69]. The correlations in economic status among American siblings (between .31 and .50) are much higher than that in Nordic countries (between 0.14 and 0.26) [2,4,70-72]. However, intergenerational mobility is higher in Canada and comparable to that of Nordic countries [66,71]. Answers to this paradox—greater intergenerational mobility in Canada than in the United States but seemingly similar developmental patterns—will have to await further research. The less-expensive post-secondary education in Canada may play an important role.

Duncan et al. [62] have emphasized the striking difference between sibling and other (best-friend, neighbor, and school mate) correlations across a range of achievement and behavior measures [62]. Even best-friend correlations were markedly lower than sibling correlations. They do, however, caution that schools and neighborhoods “may influence adolescent developmental trajectories more strongly than they affect the levels of achievement or behavior observed at any particular point” (p. 446).

Social characteristics of neighborhoods may be more important in shaping families and individuals with regard to other measures (such as crime and the perception of safety). American low-income black families experienced higher rates of adult employment and better developmental outcomes after experiencing dramatic changes in neighborhood environments [22,73]. The amount of environmental variation and the outcomes studied are likely to be critical here.

The policy implications of our findings vary somewhat according to whether means/proportions or sibling correlations are being considered. Raising mean achievement scores and reducing teenage pregnancy might respond to efforts directed toward schools enrolling higher proportions of lower income students. Lowering sibling correlations might involve efforts directed toward poorer families having one or more children of high potential. The EDI (Early Development Index) is an increasingly used tool which could help with such identification [74]. Teenage pregnancy prevention programs might also be characterized in terms of their likely impacts on affecting rates and/or sibling correlations [75].

Efforts to understand family circumstances and life events represent an important frontier in the study of outcomes and possibilities. International comparisons and additional analyses of family characteristics are called for [3]. Our goal now is to trace the path of outcomes and correlations over the course of child development. New data sets on housing and criminal justice will help broaden these efforts. More powerful research designs based on multilevel modelling will aid in this work.

## Appendix 1

Because income quintiles differ substantially in test participation, eliminating students not taking the Language Arts test changes the index scores considerably [76]. Although statistically significant at the .01 level, Q1-Q5 means for the restricted index range only from .71 (Q1) to .86 (Q5). In contrast, mean scores on the full Language Arts index vary from -0.49 (Q1) to .57 (Q5).

The restricted index also affects the sibling correlations. They go from .540 (full) to .505 (restricted) among Q1 Winnipeg residents and from .348 (full) to .302 (restricted) among their Q5 counterparts. Overall Winnipeg sibling correlations on the Language Arts index are reduced from .449 (full) to .368 (restricted); outside Winnipeg correlations drop from .575 (full) to .341 (restricted). Neighbor correlations, already very low, are further reduced by using the restricted index.

## Appendix 2. Model details

The general specification of the linear mixed model with *k* individuals in *j* subgroups, which form *i* groups is:

(1) $$Y_{\text{ijk}}=x_{\text{ijk}}^{T}\beta +v_{i}+w_{\mathrm{ij}}+e_{\text{ijk}}$$

where $x_{\text{ijk}}^{T}\beta$ are explanatory variables for each individual and coefficients, and $w_{\mathrm{ij}}\sim N\left(0,\sigma_{w}^{2}\right)$ random effects, distributed independently of $e_{\text{ijk}}\sim N\left(0,\sigma_{e}^{2}\right)$. Calculation of the intraclass correlation coefficients uses a random intercept model, omitting the explanatory variables. These are used only in the adjusted model. The intraclass correlation coefficient, *ρ*, measures the extent to which individuals in subgroup *j* in group *i* behave alike, relative to individuals across groups. Hence, the ICC (*ρ*) for the continuous variables (LA Index, Health Status and Hospital and Physician Costs) is the proportion of total variance of an observation that is associated with the class to which it belongs, and is formulated as follows:

(2) $$\rho =\frac{\sigma_{V}^{2}}{\sigma_{V}^{2}+\sigma_{W}^{2}+\sigma_{\epsilon }^{2}}$$

$\sigma_{V}^{2}$ is the variance between groups (unrelated neighbors), $\sigma_{W}^{2}$ is the variance between subgroups (siblings), and $\sigma_{\epsilon }^{2}$ the variance between individuals. Therefore, *ρ* is the proportion of total variance that can be attributed to being between groups (or subgroups if the numerator is $\sigma_{W}^{2}$).

Binary outcomes since must be modeled in a non-linear way. Following Rodríguez and Elo [77], sibling and neighborhood effects were calculated separately, generally specified in a linear mixed model as:

(3) $$Y_{\mathrm{ij}}=x_{\mathrm{ij}}^{T}\beta +v_{i}+e_{\mathrm{ij}}$$

(similar to (1)). However, given the binary nature of *Y*, the relationship is non-linear, and the realization of *Y* is conditional on the unobserved random effects *v*_*i*_:

(4) $$\pi_{\mathrm{ij}}=\mathrm{Pr}\left(Y_{\mathrm{ij}}=1|v_{i}\right)=F\left(x_{\mathrm{ij}}^{T}\beta +v_{i}\right)$$

where *F* is the standard logistic distribution cumulative density function exp(**xβ**)/(1 + exp(**xβ**)). This model can be expressed in terms of a latent variable by assuming that *Y*_*ij*_ = 1 if and only if the latent variable (Y*) is greater than some threshold value, $Y_{\mathrm{ij}}^{*}>0$. Due to the logistic distribution, *e*_*ij*_ in (3) now has a mean of 0 and variance equal to that of a standard logistic distribution ($\sigma_{e}^{2}=\pi^{2}/3$). Hence, the latent ICC using this distribution of *e*_*ij*_ is:

(5) $$\rho_{\text{logit}}=\frac{\sigma_{v}^{2}}{\sigma_{v}^{2}+\pi^{2}/3}$$

This produces correlations on the *latent* scale, which are higher than correlations calculated using dichotomous (or manifest) outcomes [77]. Hence, the ICC for binary outcomes are slightly overestimated with respect to those for the continuous outcomes. However, the main focus of this paper is to compare correlations in outcome measures across and within stratifications, which means comparing binary correlations to each other, and continuous correlations to each other, so this does not pose a major problem in the interpretation of our results.

## Abbreviations

PSID: American Panel Study of Income Dynamics; LA: Language Arts; ADG: Aggregated Diagnosis Group; MCHP: Manitoba Centre for Health Policy; Q1: Lowest income quintile; Q5: Highest income quintile; ACG: Adjusted Clinical Group; REML: Restricted maximum likelihood; ICC: Intraclass correlation.

## Competing interests

The authors declare that they have no competing interests.

## Authors' contributions

LR, JW, and RW developed the study design. RW organized the data and conducted the statistical analyses. LR and JW were the primary writers of the manuscript and all authors participated in critical revisions of the manuscript. All authors read and approved the final submitted version.

## Authors’ information

Leslie L. Roos is a Distinguished Professor in the Faculty of Medicine at the University of Manitoba. A founding director of the Manitoba Centre for Health Policy, he is among the most highly cited Canadian scientists. Les is particularly interested in the diverse uses of information-rich research environments. He is a fellow of the Canadian Academy of Health Sciences and a member of the Academy of Sciences of the Royal Society of Canada. He has been an associate of the Canadian Institute for Advanced Research and a fellow of the Academy for Health Services Research and Health Policy. Randy Walld is a Data Analyst at the Manitoba Centre for Health Policy in the Faculty of Medicine at the University of Manitoba. Randy has co-authored many highly cited publications in his more than twenty years at the University of Manitoba. Julia Witt is Assistant Professor in the Department of Economics, University of Manitoba, specializing in Health Economics. Her research covers a variety of topics with a focus on health policy, including the health workforce, inequality in health and the use of information.

## Pre-publication history

The pre-publication history for this paper can be accessed here:

http://www.biomedcentral.com/1471-2458/14/506/prepub
